# Advances in Preventive and Therapeutic Approaches for Dental Erosion: A Systematic Review

**DOI:** 10.3390/dj11120274

**Published:** 2023-11-29

**Authors:** Francesco Inchingolo, Gianna Dipalma, Daniela Azzollini, Irma Trilli, Vincenzo Carpentiere, Denisa Hazballa, Ioana Roxana Bordea, Andrea Palermo, Alessio Danilo Inchingolo, Angelo Michele Inchingolo

**Affiliations:** 1 Interdisciplinary Department of Medicine D.I.M., University of Bari “Aldo Moro”, 70124 Bari, Italy; giannadipalma@tiscali.it (G.D.); daniela.azzollini93@gmail.com (D.A.); irmatrilli@hotmail.com (I.T.); vincenzo.carpentiere@gmail.com (V.C.); denisahazballa@gmail.com (D.H.); ad.inchingolo@libero.it (A.D.I.); angeloinchingolo@gmail.com (A.M.I.); 2 Department of Oral Rehabilitation, Faculty of Dentistry, Iuliu Hatieganu University of Medicine and Pharmacy, 400012 Cluj-Napoca, Romania; 3 College of Medicine and Dentistry Birmingham, University of Birmingham, Birmingham B4 6BN, UK; andrea.palermo2004@libero.it

**Keywords:** dental enamel, tooth erosion, demineralization, erosive tooth wear, remineralization, preventive therapy, conservative therapy

## Abstract

This review discusses both preventive measures and clinically implemented therapy procedures that have been developed recently for the prevention and treatment of tooth erosion. Methods: The databases PubMed, Scopus, and Web of Science were used for a thorough search. Studies on the prevention and treatment of dental erosion that were conducted in English and used in vitro were among the inclusion criteria. Results: The search turned up 391 papers in total, with 34 of those publications matching the requirements for inclusion. Varnishes, toothpastes, and solutions containing fluoride and other substances were used as preventive measures. Conclusions: Dental erosion is a significant issue, and taking preventative steps is crucial to lessening the disease’s spread and its effects. Interventions based on fluoride seem to be successful at halting erosion and encouraging remineralization. To effectively address severe tooth erosion, therapeutic methods, including composite restorations, prosthetic crowns, and veneers, are available. Dental erosion causes aesthetic and functional issues that are best addressed with less invasive treatments like direct composite restorations. To improve and broaden the range of available treatments for this common dental issue, additional research and development are required.

## 1. Introduction

Dental erosion is a progressive, irreversible loss of dental tissue that is chemically corroded by extrinsic and intrinsic acids through a process that does not involve bacteria [1]. The condition known as “tooth wear” was described in 2019 by the International Cariology Research Group for Dental Research (IADR) and the European Organisation for Caries Research (ORCA) as the cumulative surface loss of mineralized tooth substance brought on by physical or chemical-physical processes (dental erosion, attrition, and abrasion). While “erosive tooth wear” (ETW) was described as tooth wear caused primarily by dental erosion [2,3]. Until now, dental erosion has had no clinical-scientific status, but thanks to a significant rise in prevalence in recent years, something has changed, sparking interest in dental erosion studies and research in various health disciplines, particularly in children and adolescents [4]. A European study found that an average of 29% of young adults (18–35 years) had dental erosion, and 3% showed severe signs of erosion, with significant differences between countries [5].

Both adults and children are susceptible to dental erosion [6]. It may just affect a small area of the tooth or the entire dentition [7], and its aetiology is linked to demographic changes, particularly those leading to a diet that strongly favours acidic foods and drinks [8,9]. Acids that attack dental enamel can originate from exogenous and endogenous factors [10,11]. In particular, due to its low pH, around 1 [12], the thin hydrochloric acid produced by the patient’s stomach causes the disintegration of the enamel. This happens especially in patients who suffer from gastroesophageal reflux disease (GERD), chronic vomiting, and eating disorders including anorexia and bulimia [13,14,15]. On the other hand, exogenous acids come from eating acidic meals that are either solid or liquid, like fruits, fruit juices, and carbonated beverages. When erosion comes as a result of eating disorders, it advances faster than in cases where it comes from eating or drinking acidic foods. Moreover, they may originate from drugs that include acids, such as (aspirin^®^) and ascorbic acid (vitamin C) [16,17]. On the labial sides of the maxillary anterior teeth, there are scooped-out depressions that identify the erosion structure in this instance [18,19]. Anyway, some authors also found that the origin results were unidentified because neither the anamnesis nor the examination nor the other tests enable us to determine the exact cause of the apparent lesion [16,20]. The severity of the related damage depends on the type, temperature, concentration, and length of time that the acid is in contact with the tooth surfaces, but the erosive potential is also affected by other elements like pH, ion concentrations, titratable acidity, frequency of exposure, and exposure method [21,22]. At the beginning of the erosive process, the surface layer of the enamel sees mineral loss, resulting in gradual softening of the surface. This softened layer thickness is between 0.02 and 3 μm [20]. The most superficial layer dissolves as the acid strikes and the softening process continues, and it is completely lost [22]. As was already mentioned, the minerals found in dental tissues are flawed versions of hydroxyapatite (HA). As the mineral crystals develop during the formation of hard tissue, “impurity” ions from tissue fluids are incorporated into the crystals, leading to defects. The localization of enamel prisms at the level of the amelo–cement junction makes that portion more soluble to acid attack [23,24].

Dental erosion is still a problem that needs to be addressed in terms of determining the causes, preventing it, and putting a proper treatment in place [25,26]. Promoting prevention strategies that effectively stop tooth erosion requires oral health education, dietary analysis, oral health promotion, counselling, and topical application of anti-erosion medications, which are typically used to prevent dental erosion [27,28,29,30,31,32]. Current methods of preventing this issue are focused on adhesive protocols and novel biomimetic techniques that profit from nanotechnology advancements. They consist of fillers, new bioactive polymers, mouthwash, or toothpaste (TP) that promote calcium phosphate remineralization, such as ions of calcium, phosphate, and mostly fluoride [26,33,34,35,36,37]. There are several remineralizing products on the market with different mechanisms of action, but the most effective treatment is not yet clear.

**Figure 1:**
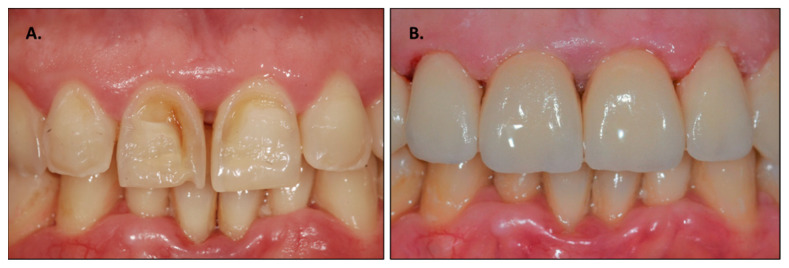
Example of anterior tooth erosion treatment using veneers: (**A**) pre-treatment; (**B**) post-treatment.

In cases where the erosion process has taken more of a hold, however, one must proceed with direct composite restorations or with composite/ceramic veneers to cover the eroded areas. Also, in these cases, it is often difficult for the clinicians to choose the best strategy to adopt as there are no guidelines [38,39,40]. Figure 1 shows an example of anterior tooth erosion resolution using veneers (Figure 1).

The purpose of this review is to report on possible and recent solutions that the clinician can implement to solve dental erosion in terms of preventive treatments and therapeutic solutions.

In particular, preventive treatment means the implementation of all measures to limit the progression of dental erosion and contain it, while restorative treatment means the conservative restoration of the dental element with anatomical restoration.

## 2. Materials and Methods

The approach for this review complies with the preferred reporting items for systematic reviews and meta-analyses (PRISMA) guidelines [41]. The review protocol was registered at PROSPERO under the unique number 460821.

The databases used for article identification were PubMed, Scopus, and Web of Science. The following keywords ‘dental erosion’ and (‘therapy’ OR ‘treatment’) were applied for the search (Table 1).

The authors checked the titles and full texts of all articles that could be relevant to the topic of the review.

Articles with these inclusion criteria were considered:

- Studies in English.
- Articles whose full text is available.
- Clinical studies and in vitro studies were performed only on human teeth.

Articles with these exclusion criteria were deleted:

- Reviews, letters, or commentaries.
- Studies in which animal enamel was used.
- Studies that did not deal with dental erosion.
- Studies that discussed only the diagnosis of the disease.

The selected studies were subdivided according to the type of intervention, whether preventive or therapeutic, and then analysed by type of design (clinical trials, case series, in vitro studies), type of subject, therapeutic strategy adopted, and outcomes obtained.

### Quality Assessment

The quality of the included papers was assessed by two reviewers, (G.D.) and (A.P.), using the ROBINS-I, a tool developed to assess the risk of bias in the results of non-randomised studies that compare the health effects of two or more interventions. Seven points were evaluated, and each was assigned a degree of bias. A third reviewer (F.I.) was consulted in the event of a disagreement until an agreement was reached.

## 3. Results

**Figure 2:**
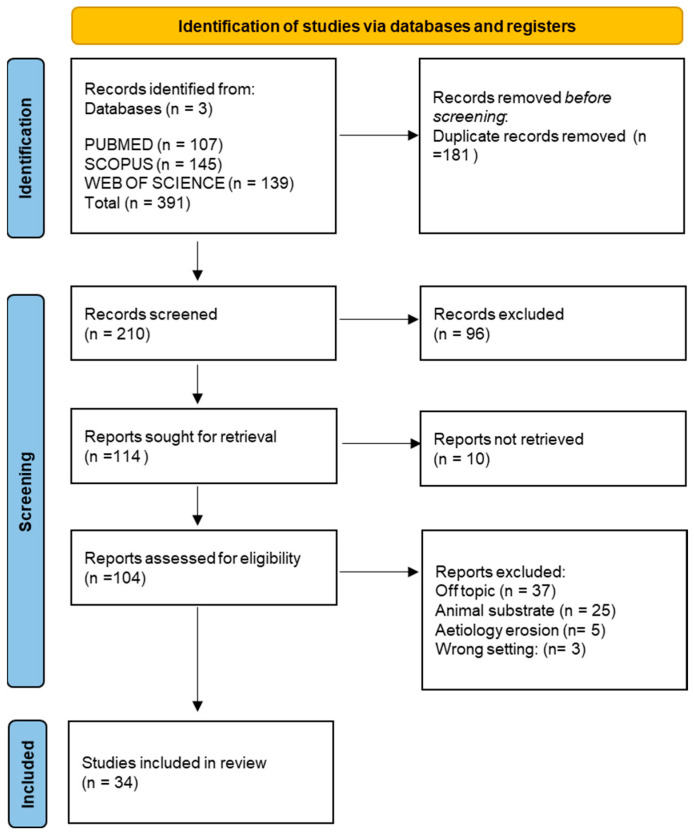
Literature search flow-chart.

The electronic search of the three databases identified a total of 391 studies. Specifically, 107 on PubMed, 139 on Web of Science, and 145 on Scopus. A total of 181 duplicates were identified. Abstract and title screenings were performed on 210 articles. Of these, 96 were excluded after checking the relevance of the topic by title and abstract. A total of 114 articles were selected for eligibility assessment, of which 10 were eliminated because the full text could not be retrieved. From the 104 reports evaluated for eligibility, five were excluded because they did not discuss the treatment, but they were studies on erosion-inducing causes. Subsequently, in total, 37 were off-topic, 25 had an animal substrate (bovine enamel), and three articles were eliminated for the wrong setting. Finally, 34 articles were selected. The selection process is summarised in Figure 2.

Furthermore, the included articles were divided into two groups: the first group dealt with all preventive methods of solving the problem (Table 2), while the second group of studies focused on its therapeutic solution (Table 3).

### Quality Assessment and Risk of Bias

**Figure 3:**
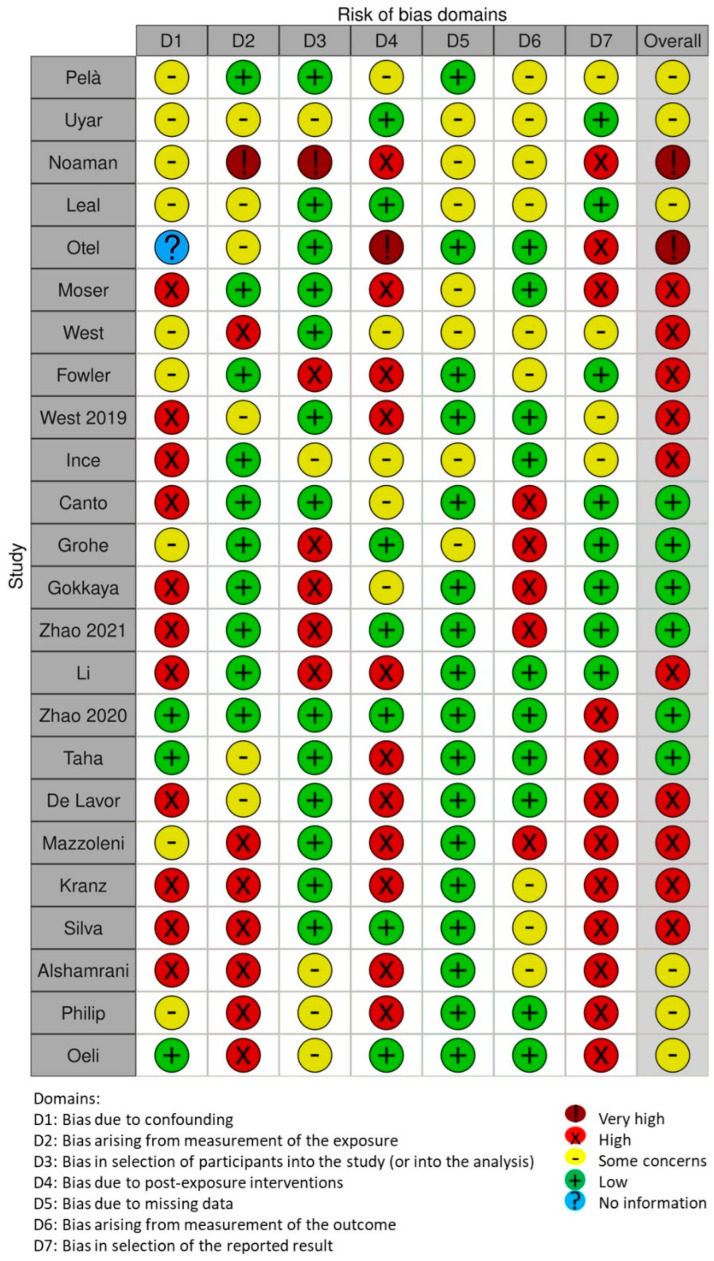
Bias assessment.

The risk of bias in the included studies is reported in Figure 3. Regarding the bias due to confounding, most studies have a high risk. The bias arising from measurement is a parameter with a low risk of bias. Most studies have a low risk of bias due to bias in the selection of participants. Bias due to post-exposure cannot be calculated due to the high heterogeneity. The bias due to missing data is low in many studies. The bias arising from the measurement of the outcome Is low. The bias in the selection of the reported results is high in the majority of studies. The final results show that 6 studies have a low risk of bias, 10 studies have a high risk of bias, 2 have a very high risk of bias, and the remainder have a questionable risk of bias.

## 4. Discussion

In recent years, the discovery and use of various methods to resolve dental erosion have produced a rich literature of in vitro and other experimental studies.

### 4.1. Preventive Therapy

Preventive measures are essential for minimising the risk of dental erosion and erosive tooth wear development. Utilising varnishes and/or TP formulations containing state-of-the-art solutions is a crucial component of preventive treatment.

A study on the prevention of erosion caused by acid from orange soda on permanent and primary teeth showed a superior efficacy of TP with fluoride over TP without floride [43]. In a similar study, deciduous teeth were subjected to the erosive action of Pepsi, and the protective effects of different gels were evaluated. The authors’ results identified NHA gel as the most effective preventive product [76]. Pelá et al. (2022) observed in vitro that the combination of sugarcane cystatin (CaneCPI-5) and sodium fluoride (NaF) was able to create a protective film on the tooth surface, preventing erosion from occurring [42]. Leal et al. (2020), in a study of 48 enamel samples, evaluated the effect of a commercial TP containing fluoride, calcium silicate, and sodium phosphate and its serum in a dual-phase gel on the prevention of erosive enamel wear. The enamel samples were divided into four groups. TPs with or without the dual-phase gel were able to prevent erosive tooth wear [45]. Several investigations have been carried out to establish the effectiveness of a combination of TP and tin (Sn2+)-containing compounds. The latter have anti-erosive properties at appropriate concentrations. However, the authors advise against the use of high concentrations of Sn2+, as adverse effects such as astringency of the teeth may occur [47].

Furthermore, West’s studies investigated the beneficial effects of a 0.454% stabilised stannous fluoride PT (SnF_2_) against other types of commercial PT. In this study of 36 volunteers, SnF_2_ TP significantly outperformed NaF/triclosan TP in terms of effectiveness in protecting against dental erosion [76]. The same authors also confirmed its action in another study using a 10-day in situ erosion model [50]. Fowler et al. (2021) observed that controlled pH 6.2 (NaF/CL) TP formulations with lactate ions and PVM/MA increased fluoride absorption and improved fluoride-mediated acid resistance. With the highest levels of fluoride and calcium absorption in all treatment groups, NaF/CL TP offered the greatest protection against tooth erosion [49]. Ince et al. compared the effectiveness of fluoride TP (1400 ppm) and TP with 1% NHA in restoring degraded enamel in situ. The results revealed that only TP with NHA significantly increased enamel surface hardness and apatite crystal deposition on the eroded surface. Fluoride TP showed no appreciable surface deposition. Chemical analysis also confirmed that tooth surfaces treated with TP and NHA had higher calcium and phosphorus content than the control group [51]. Zhao et al. (2021): The study was done on thirty-three dentin discs to evaluate the performance of a desensitising TP. A TP containing CPP copolymers and a TP with PVM/MA were used with soft brushes. These TPs can occlude dentinal tubules and resist erosive challenges. These inorganic fillers can penetrate dentinal tubules and resist erosive challenges [55].

The remineralizing impact of an anti-erosion mousse on the enamel of anterior teeth eroded by the use of a medicinal syrup was evaluated by Taha and Quasim in 2021. Forty deciduous teeth were evaluated. Simultaneous use of Elmex Erosion Protection and GC Tooth Mousse Plus showed a better remineralizing effect in the examined teeth [58].

De Lavor et al. (2021) compared several varieties of TP recommended for dental erosion with a novel TP equipped with a controlled fluoride release mechanism (NanoF). The authors aimed to determine whether the TP promotes surface remineralization in enamel erosion lesions. The researchers concluded that TP containing NanoF showed surface remineralization comparable to that achieved with sodium fluoride. NanoF TP could be an alternative for the treatment and prevention of people with dental erosion [59].

As regards varnish therapy, the inhibiting action of fluoride varnish against the effect of citric acid was analysed by Otel et al. (2022). Ten human tooth samples were evaluated in three phases: before treatment, after varnish application, and after acid attack. Fluoridated dental varnish showed a protective effect on human enamel against the dental erosion process [46].

Canto et al. (2020) evaluated and compared the effects of a single application of calcium mesoporous silica nanoparticles and other calcium and/or fluoride products in reducing the progression of tooth erosion. They concluded that Ca2+-MSN and NaF treatments can reduce roughness and loss of tooth structure. Treatments with Ca2+-MSN and NaF were superior to the negative control, with a good reduction of roughness [52]. The addition of fluoride appears to be the most frequent and effective strategy to remineralize a tooth compromised by dental erosion. For this reason, some studies deal with how to make the most of its action by reducing its concentration to avoid adverse effects [53]. In 2020, Gokkaya et al. conducted a study on 28 enamel specimens that were treated with four re-generalisation methods. Varnishes containing CPP-ACP, and fluoride prevent erosive enamel wear in teeth in vitro. CPP-ACP-containing agents have a statistically significant impact on preventing tooth erosion. In addition, CPP-ACPF-containing varnishes have the greatest effect on the remineralization process [54]. Li et al. (2022) The objective of this in vitro study was to examine how the antierosive substances quercetin, chlorhexidine, sodium fluoride, and deionized water affected dentin erosion (EDL). In summary, CHX and QUE performed best in the management of EDL. QUE gave the best results when it was used before erosive attacks, while CHX was more successful in reducing EDL when it was used after erosive situations [56]. In this in vitro investigation, Mazzoleni et al. (2023) compared the efficacy of two types of TP (one fluoride and one fluoride-free) and fluoride varnish to restore the mineral content of enamel after repeated acidification and to prevent demineralization. From this study, it appears that fluoride TPs and varnishes are good tools for surface remineralization and protection from demineralization in an acid environment [60]. On decalcified enamel surfaces, the validity of various remineralizing elements, including micro- or nanoparticles of synthetic HA, is still a matter of debate. As shown in the study by Kranz et al., the application of TP containing HA crystals on artificially demineralized dentin and enamel surfaces had only a modest and insignificant impact on dentin and no appreciable effect on enamel [61].

Dental erosion can be treated using three different methods, especially if it is discovered early enough to restore the underlying hard tissue loss. The gold standard for the prevention of tooth decay and the treatment of early carious lesions seems to be fluoride [77,78,79]. Indeed, it has been shown how fluoride varnish, applied topically in gel form, acts on enamel by exchanging the hydroxyl group into HA to form fluorapatite or fluoro-HA (more resistant to acid attack [64]), causing the precipitation of calcium fluoride, not only on the enamel but also on exposed dentinal tubules, reducing dentinal hypersensitivity [65].

In these articles, the authors evaluate the use of CO_2_ lasers and erbium and chromium lasers. The effects of titanium tetrafluoride (TiF_4_) and the applications of amine fluoride (AmF), sodium fluoride (NaF), and SnF2 alone and in combination with short-pulsed 9.3 μm CO_2_ laser irradiation against erosion in human enamel were investigated by Silva et al. (2020). By employing short-pulsed CO_2_ 9.3 micron laser irradiation and additional applications of AmF/NaF/SnCl_2_ solution, tooth enamel erosion can be greatly slowed down [62]. According to AlShamrani et al. (2021), the increased resistance of enamel to acid could lead to changes in enamel microhardness and help prevent cavitation. Topical fluoride treatment and erbium and chromium laser irradiation help dental substrates become more acid-resistant. Before the application of fluoride, laser irradiation improved the microhardness of the enamel surface, stopping its erosion [63].

### 4.2. Dental Treatment

The dentist must have the judgement to assess any potential loss of the anatomical features of the tooth, such as the dental girdle, mammelons, and/or loss of the vestibular surfaces. In line with the progression of the erosion, the patient becomes more aware of the damage that is visible at the level of some teeth, but it is important to emphasise the significance of the damages that the patient is unable to recognise: changes to the occlusion. Most of the time, teeth tend to maintain contact with opposing teeth, and because dental wear tends to be gradual, such interocclusal relationships are maintained but with a kind of excessive eruption. Everything points to there being little room for positioning potential rehabilitation projects [77]. The *European Consensus Statement on Guidelines for the Management of Severe Dental Wear* published in 2017 states that restorative intervention is only necessary if the patient presents with one or more of the following complaints: (1) sensitivity or pain, (2) impairment of function, (3) impairment of aesthetics due to loss of dental hard tissue, and (4) ‘crumbling’ of dental hard tissue and/or restorations that threatens the integrity of the remaining tooth structure [80]. It is, therefore, clear that in the presence of one or more of these problems, treatment must be resorted to, and the choice of materials to be used is far from well delineated in the literature [67,80]. On the other hand, it was well clarified by the European Federation of Restorative Dentistry in 2015 that direct restorative techniques are less invasive than indirect ones and that, as far as possible, they should be preferred in patients with severe tooth wear, along with the consideration that the use of composites should always be preferred [81]. Therapeutic rehabilitation of patients with eroded teeth consists of restorative and restorative-prosthetic treatment. There is the possibility of masking this clinical situation using dental composites, a family of aesthetic materials that allows the eroded tooth to be restored to its correct shape and size. The resin composite restorations are reasonably priced, provide good comprehensive aesthetics, and require only simple maintenance in the form of reconstruction and replacement [82,83], as demonstrated by Pini et al. in their work when the clinical follow-up at seven years has shown the maintenance of results and restorations visible [71] and in the case report by Alhammadi et al. in which a 57 year old man has been treated with direct composite restorations [74]. Additive techniques consist of the mass-mass modelling of composite materials carefully chosen based on their colour and type of filler in their respective formulation, which is done in the dental chair in a single session and which allows, after appropriate pre-treatment with 37% orthophosphoric acid etching and the use of adhesive techniques, the restoration of the tooth or teeth concerned with excellent compliance and relative costs for the patient [84,85]. In this context, the injection moulding technique, also known as the injectable composite resin technique, can be used to provide great patient compliance and enhance the aesthetics of impaired anterior teeth in certain erosive situations of the anterior sectors [67]. Such direct in-mouth restorations are, however, only subject to discoloration at the restorative-tooth interface over the years and to partial and/or total fractures with the need to return to the practice. Another therapeutic option consists in the fabrication of prosthetic crowns that embrace the eroded and suitably pre-prepared tooth structure according to specific prosthetic principles that are directly cemented in an initially provisional and then definitive manner and that guarantee a likely longer-lasting result while at the same time being a more invasive and expensive therapy for the patient [67]. If the tooth involvement is at the level of the anterior frontal sector, modern dentistry today offers the possibility of resorting to the use of aesthetic veneers that are adhesively cemented into the tooth structure that has remained minimally or not at all prepared. This prosthetic-aesthetic rehabilitation requires excellent preparation and selection of the specific case by the clinician and maximum precision in its fabrication [71].

Modern dentistry is increasingly pushing towards minimally invasive and conservative approaches, such as the ‘sandwich technique’, designed specifically for patients with a combination of vestibular and lingual erosion on the front teeth, which uses a ceramic veneer or a composite resin veneer on the vestibule and another on the palatal surface of the tooth, or other techniques, such as ‘V-shaped veneers’, as in the case treated by the University of Bologna. The latter requires only marginal preparation and, if necessary, a small correction in the middle third of the anterior teeth to achieve an incisal insertion path [70]. The work of Menezes–Silva et al. presents a possible alternative treatment, with a 24-month follow-up, to restore extensive erosive wear of gastro-oesophageal reflux teeth by restorations made with Equia Forte high-viscosity glass ionomer cement (GC Corporation, Tokyo, Japan), to restore dental anatomy and consequently reduce pain symptoms [68]. Adhesion to dentin creates a high-stress mechanical bond with a biologically active surface, and co-polymerization produces a chemical bond between the GIC and the composite resin and creates a stress-free bond at the interface of the restoration [69]. Several studies in the literature have also highlighted the potential of immediate dentine sealing in all teeth. In practice, before indirect composite and ceramic restorations are placed, sealing can optimise the adhesion of the underlying dentin by using a dentin bonding agent immediately before the impression of the tooth after its preparation [70]. In this regard, a study by Oudkerk et al. reported on experience with the one-step no-prep approach for the complete restoration of worn dentition using PICN CAD-CAM restorations. The authors included seven patients for a total of 192 restorations, all with severe wear. Vita Enamic no-prep restorations (palatal veneers and posterior restorations) were restored within 24 h after patient data were gathered. The buccal joint on the anterior teeth was covered with direct composites, and the patients then received maxillofacial physiotherapy. The restorations were assessed using the World Dental Federation’s standards. It was assessed how the treatment affected the Oral-Health-Impact-Profile-49 (OHIP-49) score. The authors reported a mean VDO increase of 5.09 × 0.85 mm on the incisal post, while the mean restoration thickness on the molars was 0.55 × 0.21 mm, and the lowest value was 0.11 mm. The fixes had a 100% 2-year survival rate and a 93.5% success rate, with 11 minor chips and 1 separation. The overall score of the OHIP-49 significantly increased [73,85].

With the improvement of research and technology, the clinician is now able to take advantage of prosthetic restorations that are optimally fabricated using traditional and/or CAD-CAM systems. In this regard, using CAD-CAM technology, Schlichting et al. compared the production of ultra-thin posterior veneers in both composite and ceramic materials. The authors demonstrated in 11 patients treated that the aesthetic-functional results and the medium- to long-term survival of the restorations proved to be the same irrespective of the material chosen for the prosthetic fabrication, although they pointed out a minimal number of partial failures and increased roughness of the composite resin compared to the ceramic surface [33].

Torosyan et al. also tried to ascertain the survival rates and technical outcomes of minimally invasive full-mouth rehabilitation in patients with erosion in a retrospective clinical analysis including 28 patients. All patient records were reviewed for a total of 406 restorations (149 direct composites, 110 onlays, and 147 veneers) supported by 365 teeth. Direct composites, onlays, and veneers all showed 6-year survival rates of 97.3%, 98.2%, and 100%, respectively. A total of 19 technical issues were present, 14 of which were partial fractures, 3 cracks, 1 wear issue, and 1 decementation [72].

A big question mark about the rehabilitative use of composite resins in cases of tooth erosion has always been directed at the rehabilitative issue of increasing the vertical dimension of the occlusion necessary as a result of significant and severe tooth wear. In this regard, the paper by Taubock et al. focused on the identification of any acceptable and satisfactory results, in aesthetic-functional terms, regarding the use of composite resin rehabilitation therapy for severe generalised tooth wear with significant loss of vertical dimension. Composites have been shown to guarantee reliability, predictability, and optimisation of the result, whether microhybrid or nano-filled. Of the two subgroups of composites, however, it is the nano-filled ones that offer less discoloration at the tooth-restoration interface, less surface roughness, and slightly better mechanical-physical characteristics than the former [82].

The work of Tian Luo et al. proposes a digital workflow that creates a virtual digital patient to measure and analyse the undamaged remaining enamel area and defect depth. A porcelain veneer, crown, and composite treatment plan were then selected for the corresponding damaged teeth based on the varying conditions of remaining enamel and tooth defect, and the design and three-dimensional printing of these models were completed. It was found that a virtual inspection of the enamel helps with acquiring the proper treatment plan objectively. The stereolithographic template successfully meets the accuracy requirements for tooth preparation while conserving the maximum amount of the tooth’s hard tissue [66].

### 4.3. Limitation

The article presents a broad overview of the problem of dental erosion, analysing both preventive and therapeutic aspects. However, it should be noted that the article has some limitations that may affect its comprehensiveness and the applicability of the information presented. The article focuses mainly on the clinical and therapeutic aspects of dental erosion, leaving out a more in-depth discussion of aspects such as epidemiology, risk factors, and diagnosis. The lack of critical assessment of the quality of the included studies and the lack of details on potential conflicts of interest could limit the robustness of the conclusions drawn from the reviewed studies. A further limitation concerns the recruitment of the selected studies, which mainly consist of in vitro experiments conducted on human teeth, so that proposals for preventive treatments are only scientific suppositions and in vivo studies are needed to verify their validity in clinical practice. Overall, despite the limitations, the article provides a useful overview of preventive and therapeutic strategies for dealing with dental erosion. However, future research and studies are needed.

## 5. Conclusions

Dental erosion remains a common problem that often poses challenges to dentists. It is essential to use effective preventive and therapeutic approaches to minimise the impact of erosion on teeth. Furthermore, if damage has already occurred, it is essential to halt the progression of the disease. Our research has highlighted the growing interest in exploring topical solutions such as paints, gels, mousses, and TP that can inhibit erosive processes. While fluoride continues to be the standard for remineralization, ongoing research is exploring new formulations of substances. When preventive measures fail to stop the progression of erosion and there is a significant loss of tooth substance that compromises the anatomy, function, and aesthetics of the tooth, there is an indication to use restorative prosthetic therapies to resolve sensitivity problems, pain, and imperfections. To further advance treatment strategies, new comprehensive, long-term clinicians are needed to evaluate the effectiveness of various preventative treatments, including fluoride varnishes, gels, and mouthwashes. These studies should focus on both children and adults to determine the most reliable preventive strategies over time. Study the performance and longevity of newer materials. These proposals are useful in creating standardised clinical guidelines for the management of dental erosion. This would help doctors make informed decisions on when to implement preventative measures and when to opt for therapeutic interventions.

## Figures and Tables

**Table 1:** Database search indicators.

Articles screening strategy	Keywords: ‘dental erosion’ AND ‘therapy’ OR ‘treatment’
	Electronic Databases: PubMed, Scopus, and Web of Science
	Language: English

**Table 2:** Enrolled studies on preventive treatment.

References	Author(s)	Years	Design Study	Subject	Intervention	Outcomes
[42]	Pelà et al.	2022	In vitro	75 Humans	5 treatment groups: Control, Elmex™ (SnCl_2_/NaF/AmF), 0.1 mg/mL CaneCPI-5, 500 ppm NaF, CaneCPI-5 + NaF (Combination).	All treatments demonstrated a protective effect on enamel against dental erosion; however, the combination of CaneCPI-5 with NaF showed greater protection
[43]	Uyar et al.	2021	In vitro	Human (45 teeth)	Three toothpaste groups: - Group-1: CPP-ACP - Group-2: Organic calendula-xylitol - Group-3: Calcium hydroxyapatite-silica	Primary teeth had statistically higher volumetric surface roughness values in Group-2 and Group 3. - Significance level: *p* < 0.05.
[44]	Noaman et al.	2022	In vitro	Human (30 sound buccal segments of primary first or second molars)	Treatment with NHA gel, HA gel, and NaF gel| - Erosive exposure to Pepsi (10 days, 60 min, 3 times daily) - Second acid beverage exposure.	10% NHA gel demonstrated better treatment and prevention of erosion caused by Pepsi Cola compared to HA and NaF gels
[45]	Leal et al.	2020	In vitro	Human (48 teeth)	Divided into 4 groups: NF, SnF_2_, CSSP, CSSP + Serum|—Cyclic experiments (3x/day for 5 days) including erosive challenge with 0.05 M citric acid, treatment with toothpaste slurries, and remineralization with artificial saliva.	Dentifrice containing calcium silicate and sodium phosphate with or without the dual-phase gel was able to prevent erosive tooth wear
[46]	Otel et al.	2022	In vitro	Human	Analysis of human enamel specimens - Three stages: 1. Before treatment 2. After varnish (treatment group) or toothpaste (control) application 3. After citric acid cycle	The use of a fluorinated dental varnish suggests a protective effect for human enamel against dental erosion demineralization process
[47]	Moser et al.	2021	In vitro	162 enamel specimens from human premolars	Treatments included a humid chamber (negative control), Elmex^®^ Erosion Protection mouth rinse (positive control), and 7 solutions with varying Sn2+ concentrations and/or containing flavouring.	Sn2+ concentrations in mouth rinses may be lowered to 200 ppm without compromising the anti-erosive properties of the solution
[48]	West et al.	2019	In vitro	36 participants (33 completed the study)	Two treatment products: 0.454% stabilized stannous fluoride dentifrice (Procter and Gamble) and a marketed dentifrice control containing 0.24% sodium fluoride and 0.3% triclosan (Colgate-Palmolive). - Intra-oral appliance with polished human enamel samples. - Participants wore the appliance for 6 h per day. - Swishing with assigned dentifrice slurry twice daily. - Swishing with 250 mL of orange juice (25 mL per minute) four times daily.	SnF_2_ TP more efficient
[49]	Fowler et al.	2021	In vitro	Human	Dentifrice formulation (NaF/CL) compared to six commercial dentifrices from European and US regulatory regions.	NaF/CL dentifrice provided the highest protection against dental erosion.
[50]	West et al.	2019	In vitro	33 humans	Two treatments: - 0.454% stannous fluoride (1100 ppm F) dentifrice (Procter and Gamble) - Control dentifrice containing 0.243% sodium fluoride (1100 ppm F) and 0.3% triclosan (Colgate-Palmolive)	Stannous fluoride dentifrice provided significantly greater protection against dental erosion compared to NaF/triclosan dentifrice.
[51]	Ince et al.	2021	In vitro	21 humans	Subjects wore a palatal appliance containing five sterilized enamel specimens. - Two test regimens: 1% nano-hydroxyapatite toothpaste and 2.25% nano-hydroxyapatite/1450 ppm fluoride toothpaste. - One control: 1400 ppm fluoride toothpaste. - Enamel specimens extraorally demineralized (4 × 5 min/day) and intraorally treated with toothpastes (2 × 2 min/day).	Home use of nano-hydroxyapatite-containing toothpaste may have a protective effect against erosion at the enamel surface.|
[52]	Canto et al.	2020	In vitro	60 human teeth	60 enamel blocks covered with different treatments: - Calcium mesoporous silica nanoparticle (Ca2+-MSN) - Casein phosphopeptide-amorphous calcium phosphate (CPP-ACP) - CPP-ACP/F-(900 ppm F-) - Titanium tetrafluoride (TiF_4_ 1%) (positive control) - Sodium fluoride (NaF 1.36%) (positive control) - Milli-Q^®^ water (negative control) - Second erosive challenge after treatment.	The Ca2+-MSN and NaF treatments were superior compared with the others and the negative control
[53]	B. Grohe, S. Mittler	2021	In vitro	Human	Enamel remineralization systems with and without fluoride.	Fluoride treatment gold standard
[54]	Gokkaya et al.	2020	In vitro	Human	CPP-ACPF varnish, TCP-F varnish, NaF varnish, and deionized water.	Agents that contain CPP-ACP have a good remineralizing effect
[55]	Zhao et al.	2021	In vitro	Human	Divided into 3 groups: Toothpaste containing CPP Toothpaste containing PVM/MA and submicron silica Regular toothpaste (Controls) - Dentin discs brushed with dentifrices for 45 strokes in 30 s. - Brushing cycle repeated after immersion in artificial saliva overnight.	Desensitizing dentifrices containing CPP or PVM/MA effectively occluded dentin tubules after brushing. PVM/MA in combination with submicron silicon dioxide exhibited stronger resistance to erosive challenges by acidic beverages
[56]	Li et al.	2022	In vitro	Human	chlorhexidine, sodium fluoride, and deionized water with quercetin.	A combination of chlorhexidine and quercetin is more efficient
[57]	Zhao et al.	2020	In vitro	Human	Subjects were randomized to receive either a Sn-containing NaF dentifrice or a conventional NaF dentifrice.	Sn-containing TP better protection
[58]	Taha et al.	2021	In vitro	Human(40 teeth)	Divided into five groups (8 samples in each group). - Group (1): Samples kept in artificial saliva. - Group (2): Samples exposed to pH cycle only. - Group (3): Samples treated with GC Tooth Mousse Plus (GC-TMP) after pH cycle. - Group (4): Samples treated with Elmex erosion protection (Elm-EP) after pH cycle.	The simultaneous use of GC Tooth Mousse Plus and Elmex erosion protection paste gave better remineralizing effect than the sole use of either GC Tooth Mousse Plus or Elmex erosion protection paste
[59]	de Lavor et al.	2021	In vitro	Human (72 teeth)	Divided into 6 groups (*n* = 12): - PC (Positive Control)—100% NaF dentifrice. - NC (Negative Control)—Placebo. - 50% nF—50% NanoF + 50% free NaF dentifrice. - 100% nF—100% NanoF dentifrice. - PN—Sensodyne^®^ ProNamel™ dentifrice. - AG—Colgate^®^ Sensitive Pro-Relief™ dentifrice.	TP with nano fluoride had good remineralizing effects
[60]	Mazzoleni et al.	2023	In vitro	8 human teeth	Samples were immersed in 5 mL of soft drink for 2 min at room temperature, then rinsed with distilled/deionized water (total of eight minutes). - Application of two different types of toothpaste, one with fluoride and one without, and fluoride varnish to the sample surfaces, followed by rinsing with demineralized water. - A second acidification cycle was carried out, and products were reapplied to assess their ability to protect against demineralization.	Fluoride TP and varnishes efficient on initial acid attack
[61]	Kranz et al.	2022	In vitro	Human	Test group (*n* = 20) treated three times a day for 3 min with a zinc carbonate-hydroxyapatite-containing toothpaste (biorepair^®^). - Gently rinsed with PBS (5 s) and stored in artificial saliva until next use. - Control group (*n* = 20) received no dentifrice treatment and was stored in artificial saliva exclusively.	Treatment with biorepair^®^ did not affect enamel surfaces as proposed. Minor mineral precipitation and a reduction in surface roughness were detected among dentin surfaces only.|
[62]	Silva et al.	2020	In vitro	Human	Specific details were provided for TiF_4_ gel and AmF/NaF/SnCl_2_ applications, CO_2_ laser settings, and erosive cycling parameters.|—Surface loss (in μm) measured immediately after treatment, after 5 and 10 days of erosive cycling with citric acid.	Combination efficient
[63]	Alshamrani et al.	2021	In vitro	60 human teeth	Specific treatments for each group: Control (C), fluoride (F), laser (L), fluoride followed by laser (F + L), laser followed by fluoride (L + F)—Specimens eroded in citric acid for 10 min	acidulated phosphate fluoride application as well as laser irradiation before fluoride application increased enamel surface microhardness and prevented the progression of enamel erosion
[64]	Philip et al.	2019	Clinical Trial	Human	fluoride gel and varnish	enamel more resistant to acid attacks
[65]	Qeli et al.	2022	Clinical Trial	176 Human	(i) First group (96 patients): Tiefenfluorid^®^ treatment applied in three appointments at 7-day intervals. (ii) Second group (80 patients): EnamelastTM treatment applied seven times at 7-day intervals.	Good results of calcium fluoride

**Table 3:** Studies included the dental treatment of dental erosion.

References	Author(s)	Years	Design Study	Subject	Intervention	Outcomes
[66]	Tian Luo et al.	2022	Case report	Human	Digital approach utilizing a stereolithographic model and virtual evaluation	Virtual inspection of the enamel helps with acquiring the proper treatment plan
[67]	Salem M. et al.	2021	Case report	Human	Injection moulding technique	Good results
[68]	Menezes-Silva et al.	2022	Case report	Human	high-viscosity glass ionomer cement	improved function
[69]	Hoeppner M.G. et al.	2019	Case report	Human	Glass ionomer cement	Improved aesthetics and function of the worn teeth.
[70]	Dallari G. et al.	2021	Case report	Human	Restoration with v-shaped veneers	Good aesthetic and functional results after 3 years
[71]	Pini, N.P. et al.	2019	Case report	Human	direct composite resin techniques	Restorations until 7 years of follow-up
[72]	Torosyan A. et al.	2021	Clinical Trial	Human	3-step technique	406 restorations with 6-year survival rates were good for all the categories
[73]	Oudkerk J. et al.	2020	Clinical trial	Human	192 restorations with direct composites	2-year survival rate of restorations was 100%
[74]	Alhammadi S. et al.	2021	Case report	Human	All direct restorations	Function improved
[75]	Schlichting L.H et al.	2022	R. C. T.	Human	11 patients restored in posterior teeth with 24 ceramic and 36 composite resin ultrathin occlusal veneers	No restorations were lost. 5 partial failures

## Abbreviations

AmF	amine fluoride
BEWE	basic erosive wear examination
Ca2+-MSN	calcium mesoporous silica
CaneCPI-5	sugarcane cystatin
CPP	casein phosphopeptide
CPP-ACP	casein phosphopeptide-amorphous calcium phosphate
CZnHA	zinc-hydroxyapatite carbonate
EDL	Dentin erosion
EGCG	Fluoride and epigallocatechin gallate
ETW	erosive tooth wear
GERD	gastroesophageal reflux disease
HA	Hydroxyapatite
IADR	International Cariology Research Group for Dental Research
NaF	Sodium Fluoride
NHA	Nano-Hydroxyapatite
OHIP-49	Oral-Health-Impact-Profile-49
ORCA	European Organization for Caries Research
PRISMA	Preferred Reporting Items for Systematic Reviews and Meta-Analyses
RCT	Randomized clinical trial
PVM/MA	polyvinyl methyl ether/maleic acid copolymers.
SnF_2_/SnCl_2_	stannous fluoride
TiF_4_	titanium tetrafluoride
TP	toothpaste
